# Alternatives to Insulin for the Regulation of Blood Sugar Levels in Type 2 Diabetes

**DOI:** 10.3390/ijms21218302

**Published:** 2020-11-05

**Authors:** Stephen C. Bondy, Meixia Wu, Kedar N. Prasad

**Affiliations:** 1 Center for Occupational and Environmental Health, Department of Medicine, University of California, Irvine, CA 92697, USA; 2 Evergreen World Healthcare Center, Garden Grove, CA 92844, USA; meixiawu1997@yahoo.com; 3 Engage Global Inc., Orem, UT 84057, USA; knprasad@comcast.net

**Keywords:** diabetes, insulin, inflammation, insulin resistance diet, oxidative stress

## Abstract

This short overview focuses on the causation and treatment of type 2 diabetes (T2D). Emphasis is given to the historical basis of understanding this disease and the background leading to emergence of the central role of insulin. The strengths of insulin administration in the treatment of diabetes are profound, but these need to be balanced against several serious shortcomings of its extended use. Some alternative approaches to T2D management are considered. Insulin is no longer considered as the first choice for type 2 diabetes, and an expanding range of new therapeutic possibilities is emerging. While these may lack the potency of insulin, at a minimum, they allow a major reduction in the intensity of insulin use. In view of the rising worldwide incidence of this disease, it is imperative to develop safe and inexpensive means of limiting its potential for impairment of normal functioning.

## 1. Introduction

### 1.1. History of Diabetes and of Insulin as a Medication

A brief history of the key steps in understanding and treating diabetes mellitus is given. Many important figures are not mentioned as this short description is intended to give an idea of the global nature of developments and of the very slow progress over the millennia that has recently become greatly accelerated. Diabetes mellitus is a disease that has long been known and, over the centuries, knowledge concerning its nature has grown gradually and incrementally. It was described in 1552 B.C. by Hesi-Ra, a famous physician/dentist of the Third Dynasty of Egypt. Charaka, the first known Indian medical sage, described the pathogenesis, symptomatology, and management of diabetes in a Sanskrit text on Ayurvedic medicine written in about 600 B.C. In about 150 A.D., Arateus, a Greek physician, gave a detailed and elegant description of diabetes, a feat paralleled around the same time by the Chinese doctor Chang Chung-Ching. Matthew Dobson, an English physician, in 1776 described the sweetness of diabetic urine as being due to a sugar. This idea was further developed a by a Scottish doctor, John Rollo, into the first recommendation of a low-carbohydrate diet for treating diabetes. The renowned French scientist, Claude Bernard, in the 19th century described the glycogenic action of the liver which is very pertinent to diabetes. Using animal experimentation, the crucial role of the pancreas in diabetes was discovered in the 1880s by Joseph von Mehring (1849–1908), a German doctor, and Oscar Minkowski, a Russian pathologist, working together. This led to the finding by Georg Zuelzer, a German scientist, that injection of pancreatic extracts could benefit diabetic patients. This was followed by the purification of insulin by a Canadian, Dr. Frederick Banting, and his team, with its direct administration to diabetic patients in 1921 [1]. This protein can now be manufactured synthetically and remains the primary treatment for diabetes.

### 1.2. Intent of This Review

The goal of this review was to present a balanced view of the utility of insulin treatment of diabetes mellitus. The growing prevalence of diabetes is mentioned, and various subtypes of diabetes are noted with emphasis being given to the type 2 adult onset variant of the disease (T2D). The probable causes of T2D are described, and the strengths and weaknesses of insulin administration, the most prevalent therapy for diabetes, are evaluated. Ways in which the potential toxicity of insulin can be minimized are discussed. Lastly, a range of alternative means of controlling T2D which allow a diminished use of insulin are enumerated. The overall purpose of this survey was to support the current view that insulin should not be considered as a first choice in T2D treatment, but rather reserved for the more intractable cases of this disease. Insulin should be used in minimal amounts needed for the effective control of blood glucose levels.

## 2. Subtypes of Diabetes Mellitus

### 2.1. Type 1 Diabetes

Type 1 diabetes is an autoimmune disease encountered primarily but not solely among the young. In this disease, the beta cells of the pancreatic islets are destroyed by an autoimmune attack and insulin can no longer be produced. This disorder has a significant genetic component in that there is a 40% concordance rate between monozygotic twins [2]. However, environmental factors also have a major potential to influence the incidence of this disease [3]. In a multicenter study in the United Kingdom (UK), the incidence of new onset of type 1 diabetes rose by around 80% during the novel coronavirus disease (COVID-19) pandemic, with evidence of severe acute respiratory syndrome (SARS)-CoV-2 infection or exposure being found present in a high a proportion of those tested [4]. Previous evidence from the related SARS-CoV-1 virus suggests that such an effect may involve viral entry into pancreatic islet cells [5].

Type 1 diabetes is often concurrent with other autoimmune diseases such as thyroid disease and celiac disease. Unlike normally protective immune responses where inflammation is terminated, autoimmunity is sustained by chronic inflammation [6]. The most effective treatment for this type of diabetes is unquestionably insulin injection, essentially replacement therapy. This has been made more convenient and accurate by the development of improved means of glucose monitoring and of insulin administration such as automated insulin delivery systems.

### 2.2. Gestational Diabetes

Gestational diabetes can develop during pregnancy in women who have no prior history of diabetes. The causes of the disease are unknown, but it is often associated with excessive weight of the mother and lack of physical activity. Approximately 2% to 10% of pregnancies can lead to the complications of gestational diabetes. The fetus being exposed to high levels of glucose can result in an excessively large baby (macrosomia). Glucose levels can return to normal after birth of the infant, but the mother is subject to an increased risk of later developing type 2 diabetes [7].

### 2.3. Type 2 Diabetes (T2D)

Insulin, via a series of kinase activations and transductions, causes the glucose type 4 transporter channels to become embedded in the cellular membrane, allowing an exponential increase of glucose entry into the cell [8]. T2D is characterized by failure of the insulin receptors to respond to insulin, thus preventing glucose uptake from the bloodstream. Later in the disease, the production of insulin by pancreatic islet cells is also curtailed. The vast preponderance of diabetes cases (95%) in the United States constitute T2D.

### 2.4. Type 3 Diabetes

Type 3 diabetes is the name sometimes given to Alzheimer’s disease associated with insulin resistance in the brain and hyperglycemia [9]. However, this nomenclature is not generally accepted.

## 3. Growing Incidence of Type 2 Diabetes Mellitus

The global prevalence of diabetes increased fourfold between 1980 and 2014 (World Health Organization). The 2017 National Diabetes Statistics Report reported that there were 30.3 million people in the United States (US) with diabetes; thus, around 27 million people in the US have T2D. The incidence of type 2 diabetes increases with age, being less than 0.2% in children under age 18 and over 25% in those over age 65. Furthermore, over 33% of the population is estimated to have a prediabetic state, which leads to a likelihood of progressing to diabetes within 10 years. Prediabetes (impaired glucose tolerance) means that blood glucose levels are higher than the normal range but not yet high enough to lead to a diagnosis of diabetes. The shift to a modern lifestyle imposing distinctive health risks, including a more sedentary time together with reduced activity, is rapidly increasing the incidence of T2D in developing countries [10].

## 4. Association of T2D with Other Conditions

The inventory of many of the suspected causes of T2D shows a clear association with several other factors. Such correlations do not demonstrate causation and, thus, evidence for a direct contributory link is necessarily more tenuous. Even when such a connecting link is very likely, the molecular mechanisms via which a factor is instrumental in provoking onset of T2D often remain opaque.

### 4.1. Diet and Weight

T2D has a significant genetic component, with the concordance rate between monozygotic twins being 72% [11,12]. However, many environmental factors are known to be significant determinants in enabling the development of the disease and determining its severity [13]. Many of these factors are interactive and reflect societal trends. These include a sedentary lifestyle with limited physical activity, as well as an unhealthy diet emphasizing processed carbohydrates and minimal fiber [14]. These habits can lead to obesity, which has a clear connection with T2D. The mechanisms via which these features can predispose to diabetes include rapid digestion and absorption of sugars, leading to surges in blood glucose levels. The consequent persistent and repeated high blood glucose level excessively challenges insulin secretion by the beta cells of the islets of Langerhans within the pancreas. The ensuing extended elevation of insulin production ultimately leads to a lower responsivity of the insulin receptors on the surface of target cells such as those in liver, muscle, and adipose tissue. The causal relation between hyperinsulinemia and insulin resistance is unclear, but the preponderance of evidence suggests that high levels of insulin initiate insulin resistance. A bidirectional effect also exists since insulin resistance can lead to hyperinsulinemia [15]. This uncertainty concerning the nature of the precipitating factor of T2DM has relevance when considering therapeutic measures. In either case, restoration of a more normal response profile is likely to be best accomplished by avoidance of insulin as an initial treatment strategy.

### 4.2. Indices of Wellbeing

Other clear associations where the exact mechanistic link to T2D remains uncertain include alcohol consumption, smoking, and sleep disruption, especially sleep apnea [13]. Psychological influences such as stress and depression, which interweave with the above, are also associated with T2D, complicating understanding of the causal chain leading to development of the disease. There may a be bidirectional link between T2D and such conditions [16].

### 4.3. Environmental Factors

The quality of the external environment can have a significant impact on the incidence of T2D. It has been estimated that ambient fine particulate matter (PM_2.5_) can lead to the onset of 3.2 million cases of diabetes annually [17,18]. Several processes could connect contaminated ambient air with a tendency to develop T2D, including chronic inflammation, elevated blood pressure, and altered autonomic tone, leading to increased insulin resistance [19]. The purity of drinking water may also impact development of T2D [3,20]. Chemical contaminants from food, plastic, and air that have been shown to affect insulin secretion and pancreatic function include persistent endocrine-disrupting xenoestrogens, pesticides, and several heavy metals [21].

### 4.4. Dementia

T2D is a risk factor for several types of dementia such as Alzheimer’s disease and vascular dementia [22]. The improper regulation of glucose levels and presence of insulin resistance, which characterize T2D, are also accompanied by accumulation of amyloid-β and hyperphosphorylated tau, which are the hallmarks of AD [23].

## 5. Role of Oxidative Stress and Inflammation in T2D

### 5.1. Oxidative Stress

Oversupply of nutrients, especially glucose, can result in enhanced rates of mitochondrial respiration. The consequent loss of efficiency results in excess production of reactive oxygen species (ROS) [24]. The ensuing oxidative stress may be a decisive factor in furthering the pathogenesis of T2D [25]. Hyperglycemia can induce ROS generation by way of several signaling pathways [26]. In turn, oxidative stress can activate various stress-related serine/threonine kinases including JNK, ERK, and p38 MAPK leading to inhibition of insulin signaling [27]. In this mutually reinforcing manner, oxidative stress within the pancreatic beta cells can lead to apoptic events and contribute to the development of diabetes [28]. Since beta cells contain relatively low levels of antioxidant enzymes [29], there is minimal protection against oxidant events in these cells.

The Nrf2/Keap1/ARE signaling pathway is the primary transcriptional regulator of intracellular redox, and, in a mouse model of diabetes, induction of Nrf2 overexpression either by genetic Keap1 knockdown or by pharmacological means can enhance sensitivity to insulin and improve disease symptoms [30]. The pro-oxidant conditions associated with the high levels of glucose found in diabetes [31] are likely to be especially damaging to the retina and may in part explain diabetic retinopathy [32].

### 5.2. Inflammation

Immune activity and extended low-grade inflammation characterize the development of T2D. The activation of the (NOD)-like receptor protein 3 (NLRP3) inflammasome by interleukin-1β is implicated [33]. A distinctive profile of gut microbiota associated with obesity and diabetes may lead to metabolic endotoxemia, thereby contributing to the low-level inflammation. The gut bacterial profile is unusually rich in Firmicutes species producing the potent inflammogen lipopolysaccharide (LPS), which can then be systemically distributed by way of the vascular system [34]. As a result of generalized inflammation, an increased number of active macrophages are present in the pancreatic islets, and the beta cells are adversely affected [35]. The chronic low-level inflammation associated with aging can further exacerbate inflammatory damage caused by T2D, especially within the vascular system [36].

## 6. Benefits of Insulin Therapy

The classical treatment of diabetes mellitus involves the administration of insulin. This is especially effective in type 1 diabetes where life expectancy after diagnosis in infancy was 1.4 years prior to the development of insulin, but is now close to normal values. Insulin can generally rapidly lower blood glucose and additionally protects against the many adverse consequences of hyperglycemia in a range of disorders unrelated to diabetes [37,38]. Despite its shortcomings, insulin has unquestionably improved and extended a great many lives, and it continues to be a major drug of choice in many cases of diabetes [1].

The difficulty of maintenance of glucose levels within a desirable range has been very problematical with insulin. However, many advances have been made to prevent the large fluxes in glucose levels that were common in diabetics following acute administration of insulin. The modes of continuously monitoring plasma glucose and the administration of precise doses of insulin in a gradual manner have been continuously improved [39,40]. Nevertheless, effective control of blood glucose content by insulin remains imperfect and still represents a challenge.

## 7. Shortcomings of Use of Exogenous Insulin

### 7.1. Insulin Resistance

Metabolic syndrome comprises a series of associated risk factors that include obesity, insulin resistance, and hypertension. This common assembly of factors is clearly linked to an increased risk of developing type 2 diabetes, as well as atherosclerosis. Weight gain and reduced physical activity associated with metabolic syndrome lead to insulin resistance. This blocks the ability of insulin to increase tissue uptake of glucose in a normal manner and leads to hyperglycemia and decreased glucose utilization. Elevated levels of free fatty acids resulting from resistance of adipose tissue to insulin can further aggravate insulin resistance in a reciprocally potentiating manner [41]. Pharmacological reduction of levels of circulating free fatty acids results in re-sensitization of tissues to insulin and inhibits progression of atherosclerotic changes [42]. Compensatory hyperinsulinemia can initially develop in response to insulin resistance, and this in itself can promote inflammatory events. It has been shown that reducing levels of circulating insulin by immunodepletion can directly diminish inflammation [42].

Excessive levels of insulin can initiate other pathological states such as increased levels of procoagulant factors and increased sympathetic nervous activity. Later, as the β cells fail to compensate for the ongoing insulin resistance, cellular uptake of glucose is impaired and plasma glucose levels rise [43].

Disruption of normal signaling can take place at several sites related to the insulin receptor and the many kinases that it activates. High circulating levels of glucose also directly depress the effectiveness of the insulin response [44]. Insulin resistance commonly predates the onset of diabetes and is often found in unaffected first-degree relatives of diabetics [45]. This suggests a genetic predisposition and that insulin resistance can be a precipitating factor in initiating diabetes. The heightened intensity of systemic oxidative, nitrosative, and inflammatory processes concurrent with metabolic syndrome can then promote pancreatic β-cell dysfunction as discussed above. In this manner, elevated levels of glucose and depressed responsivity to insulin mutually exacerbate each other. Insulin treatment can not only lead to desensitization of insulin receptors but can also cause increased appetite and weight gain [46] and worsen insulin resistance [47]. Overall, the evidence suggests that hyperinsulinemia can be both a result of and a driver of insulin resistance [48]. While, in the short term, insulin administration is very effective, this implies that its extended use is likely to have adverse consequences.

### 7.2. Mortality and the Extent of Insulin Use

A significant dose-response association between mortality and the intensity of the extent of insulin treatment has been found in T2D patients. Even when a multivariable correction was taken into account, this relationship persisted [49]. The quality-adjusted life years index of patients using insulin is reported as being lower than that of those using metformin, and this is also evident in subjects switching from metformin to insulin [50]. A review of the effects of insulin therapy for T2D on cardiovascular health and longevity was conducted using data from outcomes trials, meta-analyses, and patient registry studies. Injected insulin led to increased cardiovascular risk and worsened mortality, perhaps instigated by weight gain and recurrent hypoglycemia [51]. The authors concluded that use of injected insulin predisposes to inflammation, atherosclerosis, hypertension, dyslipidemia, heart failure, and arrhythmias, showing that insulin therapy has an overall poorer safety profile than that found with several other therapies used to treat T2D. This is not an isolated report, as other similar ones can be found [52].

Insulin injection can also lead to several types of localized lesions, including lipoatrophy, cutaneous lipohypertrophy, allergic reaction, and insulin-derived amyloidosis [53].

## 8. T2D Treatment without Use of Exogenous Insulin

In recent years, a large range of therapeutic options and lifestyle choices for the management of T2D have been recommended. These are too many to enumerate here, but some especially promising strategies are briefly described.

### 8.1. Behavioral and Dietary Changes

These are the most simple and effective ways to attenuate the symptoms of T2D, but they are also very challenging. Simply put, much of T2D could be effectively controlled by reversal of weight gain and restoration of normal weight. This could be furthered by dietary management. Intermittent fasting is an effective means of achieving this [54,55].

The relationship between muscle activity and levels of circulating glucose was first noted in 1887 by Chauveu and Kaufman who reported that “when a horse chews on hay, the concentration of glucose in the blood draining its masseter muscle substantially decreases” [56]. Since then, evidence has grown showing the positive role of physical activity in maintaining insulin sensitivity and in reversing insulin resistance. Regular exercise is useful in reducing levels of inflammatory indices found in T2D [57]. Furthermore, these behavioral changes have several other valuable effects on cardiovascular health. The usefulness of a diet low in carbohydrates and oriented toward more fats (“ketogenic diet”) in the treatment of T2D is becoming increasingly recognized. Its value lies not only in preventing food-induced surges in blood glucose but also in promoting increased levels of circulating ketone bodies such as d-β-hydroxybutyrate (βHB) after their production by fermentation in the intestine [58]. Such ketone bodies are able to directly protect against diabetes-related vascular disease [59]. It is important to emphasize that the concentration of ketones in persons on such as diet is far lower than the toxic concentrations seen in diabetic ketoacidosis [60]. Germ-free mice without intestinal bacteria do not develop insulin resistance or weight gain when fed a high-carbohydrate high-fat diet [61]. This reveals the importance of the specific bacterial profile of microbiome in determining disease progression.

Butyrate and propionate suppress weight gain in mice with high-fat diet-induced obesity, and acetate has been proven to reduce food intake in healthy mice [62]. The nature of the lipid components of the ketogenic diet is critical in optimizing insulin sensitivity, as monounsaturated fats from fish oil promote this, while saturated fats from animal sources have the opposite effect [63]. The quality of the carbohydrate constituent of food is also important, as diets favoring vegetables, nuts, peanut butter, and whole-grain breads have been associated with lower mortality [64].

### 8.2. Pharmacological Treatment of Diabetes

A recent approach to T2D is to initiate treatment with oral medications for glycemic control. A wide range of such drugs are available with a diverse range of targets [65]. Insulin therapy is generally only initiated after the failure of one of these agents or several in combination. Two main approaches to pharmacological therapy are utilized. The first of these involves the use of drugs which seem to be antiglycemic and possess a relatively well-defined mechanism of action. This includes most of the agents referenced in detail by Chaudhury et al. (2017) [65] and some newly developed precision-focused strategies, four of which are described below.

#### 8.2.1. MicroRNA (miRNA)

miRNAs are increasingly recognized as key determinants of health or disease. Downregulation of miR-155 and miR-146a is found in T2D [36], and a role for miRNA-146 in the pathogenesis of T2D is further suggested by the report that streptozotocin-induced diabetes results in downregulation of miR-146a in rat aorta [66]. The development of insulin resistance in cardiac tissue causes reduced glucose uptake and increased fatty acid uptake and leads to accumulation of toxic fatty acids. This transition appears to be facilitated by miRNA [67]. In mice subjected to a high-fat diet, inhibition of the heart-specific microRNA, miR-208a, with an antisense oligonucleotide leads to resistance to obesity induced by a high-fat diet and re-establishes systemic insulin sensitivity and glucose tolerance [68]. This may ultimately lead to a novel strategy for T2D treatment.

#### 8.2.2. Sirtuin Activation

The sirtuins are a family of NAD(+)-dependent deacetylases that detect cellular energy availability and adjust metabolic processes. Their activation by pharmacological means can elicit multiple metabolic benefits that protect mice from diet-induced obesity, type 2 diabetes, and nonalcoholic fatty liver disease [69]. When activated, both SIRT1 and SIRT3 reduce inflammation, oxidative stress, and pancreatic damage, while promoting the maintenance of glucose homeostasis [70]. As well as improving disease resistance, characteristic aging processes can be delayed and longevity can be extended by activation of these signaling proteins [71]. The stimulation of several sirtuins appears to be beneficial for diabetes and can be specifically effected by rapamycin inhibition of mTOR [25] or rather less selectively by resveratrol [72].

#### 8.2.3. Glucagon-Like Peptide-1 Receptor Agonists

Synthetic glucagon-like peptide-1 receptor agonists can improve glycemic control with a minimal risk of hypoglycemia, and they offer another possibility of treating T2D with reduced levels of insulin. Another advantage of these agents is that some of them only require administration once weekly [73].

#### 8.2.4. Inhibitors of the Sodium-Glucose Co-Tranpsorter-2 (SGLT2)

Another valuable class of drug with a specific mechanistic focus constitutes sodium-glucose co-transporter-2 inhibitors. These block the transporters responsible for roughly 90% of the resorption of glucose from the kidney tubules. Thus, excess glucose is excreted in the urine, keeping blood levels low without stimulating insulin release. Other advantages of this type of treatment include the fact that the drugs can be taken orally and that they act independently of pancreatic β-cell function [74].

### 8.3. Broadly Acting Materials

Another class of agents constitutes those that are likely to have broad range of action on several parameters. These include phytochemicals and modern drugs originally derived from botanical sources. In the skeletal muscle of diabetic patients, consumption of cocoa, rich in catechins, leads to activation and translocation of key transcriptional factors and increases in antioxidant enzyme (superoxide dismutase and catalase) activity [75]. Metformin, originally derived from the plant *Galega officinalis* (French lilac or goat’s rue), is a widely used and very effective antihyperglycemic agent [76]. It is broadly antioxidant and anti-inflammatory, has protective effects against a range of diseases including cancer and obesity, and is likely to have several modes of action [77]. Many phytochemicals that may have usefulness in T2D treatment such as berberine, curcumin, quercetin, and resveratrol, rather than acting on a single defined target, probably have many intracellular targets and involve a range of signaling pathways [78].

An advantage of the use of materials possessing a multiplicity of sites of action or the use of more than one pharmaceutical agent is a reduced risk of counteraction of a useful effect by systemic homeostatic efforts to reverse this.

### 8.4. Treatment of T2D with Antioxidant and Anti-Inflammatory Agents

Classical antioxidant supplements have not proven markedly effective in arresting the progression of T2D [79,80,81]. However, vitamin C in combination with a flavanone glycoside, naringin, has been reported to ameliorate a streptozotocin-induced animal model of diabetes [82]. Lipoic acid administration has a similar effect on this animal model [83]. A clinical study involving treatment of T2D patients with a mixture of lipoate, carnosine, and thiamine reduced levels of circulating glucose [84]. T2D patients specifically possessing the haptoglobin Hp-2-2 genotype benefit from vitamin E supplementation [85]. It may be that supplementation with multiple antioxidants is able to reduce insulin requirement in T2D patients. The selective targeting of the critical Nrf2/Keap1/ARE antioxidant pathway which leads to expression of several antioxidant genes allows a wide range of protective enzymes to be induced. Specific Nrf2 activators include catechins, curcumin, sulforaphane, resveratrol, and pterostilbene [86]. Plants contain a multitude of phenolic compounds, and this may account for the finding that dietary antioxidants within food may be more useful than administration of synthetic preparations of common vitamins in ameliorating diabetes-related oxidative damage [32,87].

## 9. Conclusions

Some of the tactics available for the remediation of type 2 diabetes are summarized in Table 1. There is considerable overlap in these differing means of treatment, but an ever-expanding range of resources is becoming available. While each approach in isolation may have limitations, the simultaneous application of several components of the collection of resources available can be integrated. Such a combination is likely so lead to synergistic effects, thereby providing an optimal outcome.

**Figure 1:**
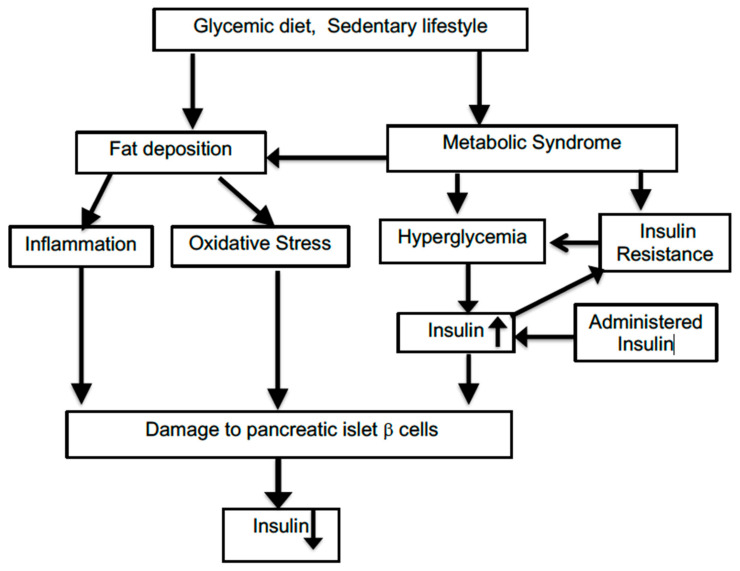
Connections among factors impacting on type 2 diabetes.

T2D is a more complex multifactorial disorder than type 1 diabetes, and it involves a series of interactive pathological changes which can be bidirectional and, thus, mutually reinforcing. The initiating trigger of such an assembly of changes can be difficult to pinpoint. Figure 1 illustrates some of the interactions among relevant factors. Many risk factors for incurring T2D and associations with T2D prevalence have been identified, but this has not been translated into a true causal understanding of the disease. As a consequence, it is difficult to model this gradually developing disease with animal systems which use relatively rapidly acting drugs such as streptozocin to disable the insulin-producing beta cells of the islet cells within the pancreas. As with many other animal models of slowly progressing human disorders such as Alzheimer’s disease, a successful reversal of abnormal features in laboratory animals cannot often be replicated by corresponding improvements in the clinical situation. T2D development has many unknown facets compared to the effects seen following a single-site chemical lesion in animals.

Obviously, initial treatment of T2D should be attempted with the least intrusive means possible. Many such measures involving lifestyle modification are available for T2D. Dietary modifications and regular exercise appear straightforward but are, in fact, very challenging. Furthermore, they do not give the impression of relying on medical expertise and may, thus, be less convincing to patients. Nevertheless, success in this area could substantially reduce the degree of reliance on antihyperglycemic agents. Another approach minimally disrupting normal metabolism may be the use of dietary supplementation with a mixture of micronutrients known to be of use in the control of blood glucose levels. This could at least reduce the insulin dose required in order to maintain glucose levels in the normal range [88]. Again, the existence of such nonprescription agents may limit the attention given by both physician and patient. This suggests that a more intense involvement of clinicians in the everyday life pattern of their patients would be helpful. Unfortunately, a means of recognizing and rewarding such a comprehensive and continuing style of medical guidance and intervention is lacking. Current medical practice emphasizes the diagnosis and treatment of a specific disease. This approach is tightly focused, and many factors that are present in the penumbra of the disorder tend to be regarded as extraneous and not crucial for therapy. Type 2 diabetes illustrates how this narrow outlook can be unsuitable and that a more holistic approach to a clinical problem considering “peripheral” factors is needed. A broader view of the setting in which the disease presents can help to establish the nature of the relevant processes preceding the frank appearance of disease. The current style of medicine in the United States accentuates the need for a rapid throughput of patients without minimal discursion into secondary issues. Remedying this perhaps by modifying the reward system of medical remuneration and applying a more integrative style of patient care could improve health outcomes substantially but would require a fresh perspective on the mode of practicing medicine.

## Figures and Tables

**Table 1:** Approaches toward management of type 2 diabetes. SIRT, sirtuin.

Goal of Treatment	Mechanism Involved	Treatment
Prevent hyperglycemia	Lower levels of circulating glucose	Insulin
		Inhibition of the renal sodium-glucose co-transporter-2 (SGLT2)
		Glucagon-like peptide-1 receptor agonists to stimulate insulin release
	Reduced rate of carbohydrate absorption	Diminished consumption of refined or simple carbohydrates
	Reduced hepatic gluconeogenesis, increased glucose uptake by muscle	Metformin
Reduction of levels of inflammation and oxidative stress	Increased antioxidant and anti-inflammatory milieu	Activation of Nrf/KEAP/ARE pathway
		Antioxidant vitamins (e.g., lipoate, α-tocopherol, and ascorbate)
		Use of phytochemicals with a broad range of properties (e.g., curcumin, resveratrol, and catechins)
		Ketogenic diet
	SIRT activation	Rapamycin
Moderation of fat deposition	Lower accumulation of fat	Physical activity
		Blocking of miR-208a with an antisense oligonucleotide
	Modulation of gut biome	Ketogenic diet
